# Prevalence, patterns and factors associated with spirometry abnormalities among children and adolescents with sickle cell disease in northern and eastern Uganda: a cross-sectional study

**DOI:** 10.3389/fped.2026.1891576

**Published:** 2026-08-28

**Authors:** Gabriel Kakuru Shamavu, Martin Nduwimana, Banga Mseza, Bienfait Mumbere Vahwere, Theoneste Hakizimana, Joshua Muhumuza, Walyeldin Elnour Elfakey, Grace Ndeezi

**Affiliations:** 1 Department of Pediatrics & Child Health, Faculty of Clinical Medicine and Dentistry, Kampala International University, Bushenyi, Uganda; 2 Department of Surgery, Faculty of Clinical Medicine and Dentistry, Kampala International University, Bushenyi, Uganda; 3 Department of Obstetrics and Gynecology, Faculty of Clinical Medicine and Dentistry, Kampala International University, Bushenyi, Uganda; 4 Department of Pediatrics, University of Bahri, Khartoum, Sudan; 5 Department of Pediatrics and Child Health, Makerere University College of Health Sciences, Kampala, Uganda

**Keywords:** pediatric, pulmonary function, sickle cell anemia, sickle cell disease, spirometry patterns, Uganda

## Abstract

**Background:**

Abnormal spirometry indices are known to be early markers of long-term respiratory dysfunction in the sickle cell disease (SCD) population. In Sub-Saharan Africa, however, evidence remains scarce. This study aimed to assess the prevalence, patterns, and factors associated with spirometry abnormalities among children and adolescents with homozygous SCD in Uganda.

**Methods:**

A cross-sectional study was conducted between september and december 2025 at Jinja and Lira Regional Referral Hospitals. Clinically stable children aged 6–18 years with confirmed homozygous SCD were consecutively enrolled. Sociodemographic and clinical data were collected using structured questionnaires, and spirometry was performed and results were interpreted using the Global Lung Initiative 2012 reference equations (GLI-12). Logistic-regression models identified factors independently associated with spirometry abnormalities.

**Results:**

Among 362 participants (mean age 11.7 ± 3.2 years; 53.1% male), 41.7% had abnormal spirometry. The restrictive spirometry pattern predominated (67.5%), followed by obstructive (27.8%) and mixed (4.6%) patterns. The odds of abnormal spirometry increased threefold among children 8–12 years (aOR = 3.346; 95% CI 1.375–8.142; *p* = 0.008) and rose over thirteen-fold in adolescents 13–18 years (aOR = 13.532; 95% CI 5.687–32.201; *p* < 0.001). Being underweight was associated with a four-fold higher risk (aOR = 3.871; 95% CI 2.189–6.845; *p* < 0.001). Children who had not completed all recommended pneumococcal-conjugate-vaccine (PCV) doses had a 3.5-fold greater risk (aOR = 3.474; 95% CI 1.618–9.153; *p* = 0.022). A history of recurrent acute chest syndrome was also associated with spirometry abnormalities (aOR = 3.295; 95% CI 1.016–14.526; *p* = 0.044).

**Conclusion:**

Spirometry abnormalities were highly prevalent among children and adolescents with SCD in Northern and Eastern Uganda, with a predominance of restrictive spirometric patterns. Increasing age, undernutrition, incomplete pneumococcal vaccination, and previous ACS episodes were independently associated with abnormal spirometry. These findings support the integration of routine spirometry testing and surveillance into pediatric SCD care in the clinical setting.

## Introduction

Sickle cell disease (SCD) is one of the most extensively studied genetic disorders and was the first human illness described at the molecular level (1). Despite advances in comprehensive care, SCD remains a major global public -health challenge. The median life expectancy for affected individuals is approximately 55 years, which is substantially lower than that of non-SCD populations (2, 3).

Respiratory complications, including chronic lung disease and pulmonary hypertension, are the leading causes of morbidity and premature mortality among individuals with SCD (4, 5). Pulmonary involvement has been recognized since Herrick’s first description of the disease in 1910 (6). Recent reports indicate that pulmonary complications account for 20%–30% of SCD-related deaths (7). While acute chest syndrome (ACS) is widely acknowledged as a life-threatening manifestation, the burden of chronic respiratory dysfunction remains under-investigated (8). Children and adolescents with SCD frequently develop chronic respiratory disorders such as asthma, sleep-disordered breathing, and reduced exercise tolerance (5, 9).

More importantly, other long-term pulmonary sequelae such as SCD-related pulmonary hypertension and pulmonary fibrosis, have been identified as long-term morbidity and mortality factors (10, 11).

These chronic complications are now understood to evolve from early, often asymptomatic reductions in lung volumes during childhood, which are detectable through spirometry (5).

Emerging evidence underscores the role of abnormalities in pulmonary function tests (PFTs) as early, objective indicators of chronic lung disease development (5, 12).

Since the 1970s, studies across different regions have consistently demonstrated a higher incidence of abnormal lung function among individuals with SCD than among their healthy peers (13). Abnormal spirometry indices serve as early objective indicators of evolving chronic lung disease (12).

Sub-Saharan Africa (SSA) bears approximatively 75%–80% of the global SCD burden, with 50%–60% of affected children dying before age five mainly due to delayed diagnosis and limited access to care (11). Survivors often develop progressive end-organ complications that impair their quality of life (12, 14). However, evidence on chronic morbidity, particularly lung function impairment among African children with SCD remains scarce (15, 16).

In Uganda, approximately 15 000 infants are born with SCD each year (17). Only one previous single-center study, conducted in Kampala by Aol et al. (15), reported a 37.9% prevalence of abnormal Lung Function among 332 urban children with SCD, but did not represent rural or high-burden regions (15). Other studies have shown variable prevalence rates from 23%–29% in Brazil and Bahrain to 25% in the United States (18–20), and as high as 62% in the study by Cook et al. (21) in Malawi, reflecting differences in environment, nutrition, and healthcare access (21).

Given the absence of nationally representative data, this study aimed to determine the prevalence, patterns, and factors associated with spirometry abnormalities among children aged 6–18 years with confirmed homozygous form of SCD (HbSS) attending Jinja and Lira regional referral hospitals in eastern and northern Uganda, which encompass both urban and rural populations with a high SCD burden.

## Methods

### Study design and setting

This health facility-based cross-sectional study employed a quantitative approach to determine the prevalence, patterns, and factors associated with spirometry abnormalities among children and adolescents with SCD attending two tertiary hospitals in Uganda. The study was conducted between september 3 and december 18, 2025 at Jinja regional referral hospital (JRRH) in the eastern region and Lira regional referral hospital (LRRH) in the northern region. These sites were purposely selected to capture regional variations in SCD burden, environmental exposures, and socioeconomic conditions.

The JRRH is a 600-bed government hospital serving approximatively 4.5 million people across 12 districts. It includes a children’s hospital that admits approximatively 6,500 patients annually and operates specialized sickle-cell clinics twice weekly (mondays for children < 12 years and wednesdays for adolescents and adults).

The LRRH is a 401-bed tertiary facility serving approximately 2.5 million people in the Lango sub-region, nearly half of whom are aged below 14 years. Its sickle-cell clinic runs weekly on Fridays. Both hospitals are internship training centers with accredited laboratories for hematology and clinical chemistry analysis. Including these two centers ensured the representation of both urban and rural SCD populations.

### Study population and sample size

The target population comprised children and adolescents aged 6–18 years with confirmed homozygous SCD (HbSS) attending outpatient sickle-cell clinics at the two selected regional referral hospitals during the study period. The sample size was calculated using the Kish and Leslie (1965) (44) formula for prevalence studies, assuming a 37.9% prevalence of abnormal lung function from results of a previous study conducted in Kampala by Aol et al. (15), a 5% margin of error, and 95% confidence level (15).

The minimum required sample size *n* $=\frac{Z^{2}p(1-p)}{e^{2}}$

Where, p: The Proportion of the characteristic of interest in the population (37.9% impaired lung function).

e: The desired margin of error, set at 5% (0.05).

z: The Z-score corresponding to a 95% confidence level, which is 1.96.

Substituting the values: $n=\frac{{\left(1.96\right)}^{2}\times 0.379\times (1-0.379)}{{\left(0.05\right)}^{2}}=361.66\approx 362$

The minimum required sample size was 362 participants, which was sufficient for the analysis of associated factors. Participants were consecutively enrolled until the target sample size was reached.

### Eligibility criteria

Children and adolescents were eligible for inclusion if they were aged 6–18 years and had confirmed homozygous SCD (HbSS), as verified by characteristic clinical features and hemoglobin electrophoresis. Participants were recruited from the sickle-cell clinics at the two selected regional referral hospitals during the study period. Only children in a stable clinical state were enrolled. Steady state was defined as having no acute painful episode requiring hospital admission within the previous four weeks, no blood transfusion in the past four months, and no intercurrent illness, such as infection or inflammation during the preceding four weeks. Children were excluded if they had chronic respiratory diseases such as asthma, pulmonary tuberculosis, or cystic fibrosis, or if they had other comorbidities that were likely to affect lung function or spirometry performance, including HIV infection, chest or spinal deformities, or neurodevelopmental disabilities. Patients with a history of thoracic surgery within the previous two years were also excluded. Participants whose parents or guardians did not provide written informed consent or who declined assent were not enrolled.

### Data collection procedures

Prior to enrolment, the eligibility of each participant was assessed according to predefined inclusion and exclusion criteria. After confirmation, written informed consent was obtained from the caregivers and assent from children aged ≥7 years, following ethical guidelines.

Data were collected using a structured, pre-tested questionnaire translated into Lusoga and Langi. Trained research assistants conducted face-to-face interviews under the principal investigator’s supervision. The questionnaire recorded each participant’s sociodemographic characteristics (age, sex, residence, parental education, and household income), clinical history (immunization and nutritional status, previous hospitalizations, transfusion history, and acute complications such as ACS or stroke), and respiratory symptoms (chronic cough, wheezing, and sleep-related breathing issues).

After the interview, the participants underwent a comprehensive physical examination, including vital signs, anthropometry, and peripheral oxygen saturation. Weight and height were measured without shoes, and body mass index (BMI) was calculated as kg/m². Nutritional status was categorized using the World Heaalth Organisation (WHO) 2009 Child Growth Standards: underweight (< −2 SD), normal (−2 to +2 SD), and overweight (> + 2 SD).

### Spirometry testing and interpretation

During the study, a total of 379 children attempted spirometry. Of these, 11 children (2.9%) were unable to complete the test due to an inability to perform the maneuvers correctly, and 6 tests (1.6%) were subsequently excluded from the final analysis due to invalid flow volume or volume-time curves that did not meet the acceptability and repeatability criteria. Consequently, 362 acceptable and repeatable tests were included in the final analysis.

Pulmonary function was assessed using spirometry. This non-invasive testing was performed in strict accordance with the 2019 ATS/ERS guidelines for equipment performance, calibration, hygiene, patient preparation, maneuver quality, and result interpretation (12, 24, 26).

All tests were conducted using a digital spirometer (CONTEC SP100B), capable of measuring Forced Expiratory Volume in one second (FEV1), Forced Vital Capacity (FVC), and FEV1/FVC ratio.

The device was calibrated daily using a 3-L syringe cycled at least three times to generate flows between 0.5 and 12 L/s with injection times of 0.5–6 s, as recommended by the 2019 ATS/ERS guidelines. When a disposable in-line filter was used, it was applied during calibration verification to ensure accuracy and consistency. Weekly biological quality control was conducted using a healthy volunteers to confirm device stability within ±5% of baseline values.

All spirometry tests were performed by the principal investigator, who completed two months of supervised spirometry training at Mbarara Regional Referral Hospital. Competency was confirmed before data collection, and all spirometry recordings were independently reviewed by two consultant pulmonologists to ensure quality and consistency. Before each test, the participants’ identity, age, sex, and ethnicity were verified, and their height and weight were measured in triplicate using calibrated instruments. Recent physical activity, medication use, and respiratory symptoms were reviewed, and the procedure was carefully explained to ensure understanding.

Spirometry was performed in the seated position using a nose clip and a tight mouth seal around a sterile, single-use mouthpiece. Each maneuver began with maximal inspiration, followed by rapid, forceful, and continuous expiration until the end of forced expiration. For children under 10 years of age, the minimum exhalation time criteria were adjusted according to the ATS/ERS pediatric recommendations.

Participants performed at least three acceptable and repeatable maneuvers, with a maximum of eight trials if necessary to meet the quality criteria. The best FEV₁ and FVC values were used for analysis, with repeatability confirmed within ≤150 mL, following ATS/ERS 2019 guidelines. The participants were tested in an upright position with a nose clip and a tight mouth seal around the mouthpiece to prevent leaks.

All results were assessed in real-time for acceptability and repeatability. Incomplete efforts or trials with cough or early termination were discarded and repeated. Rigorous infectioncontrol measures were applied, including single-use mouthpieces, gloves, and disinfection of equipment surfaces after each test.

In the absence of validated Ugandan reference equations, spirometry was interpreted using Global Lung Initiative (GLI-2012) reference equations for Black African-American populations, generating age-, sex-, and height-adjusted Z-scores; with demonstrated acceptable performance in African pediatric populations (22, 23). The results were interpreted using the Global Lung Initiative (GLI-2012) reference equations for Black African-American populations, generating age-, sex-, and height-adjusted Z-scores.

Abnormal spirometry results was defined using the Lower Limit of Normal (LLN) as a Z-score <−1.64 (<5th percentile) for FEV₁, FVC, or FEV₁/FVC. All spirometric patterns (normal, obstructive, restrictive, and mixed) were classified according to the ATS/ERS interpretive strategies (12, 24, 26). The pulmonary assessment in this study was based on spirometry indices only, not complete pulmonary function testing. Advanced assessments, such as total lung capacity measurement, diffusing capacity for carbon monoxide, and plethysmography, were not performed. Therefore, restrictive spirometry pattern should be interpreted as suggestive of possible restriction rather than confirmed restrictive ventilatory impairment. Details on the interpretation of spirometry indices and patterns are presented in Table 1.

**Table 1:** Spirometry interpretation based on the 2021 ERS/ATS technical standards and the GLI-12 equation.

Indices	Patterns	Definition
FEV1	Normal	5th to 95th percentile (−1.645 to +1.645 z-score)
	Low	< 5th percentile or < −1.645 z-score (LLN) Mild: −1.65 to −2.5SD Moderate: −2.51 to −4.0SD Severe: < −4.1SD
FVC	Normal	5th to 95th percentile (−1.645 to +1.645 z-score)
	Low	< 5th percentile or < −1.645 z-score (LLN) Mild: −1.65 to −2.5 z-score Moderate: −2.51 to −4.0 z-score Severe: < −4.1 z-score
FEV1/FVC ratio	Normal	5th to 95th percentile (−1.645 to +1.645 z-score)
	Low	< 5th percentile or < −1.645 z-score (LLN)
Spirometry-defined Lung Function	Normal	FEV1, FVC and FEV1/FVC: 5th to 95th percentile (−1.645 to +1.645 z-score)
	Restrictive spirometry pattern	FVC < LLN with FEV1/FVC ratio > LLN
	Obstructive spirometry pattern	FEV1/FVC ratio < LLN
	Mixed spirometry pattern	FVC and FEV1/FVC ratio < LLN

FEV1, forced expiratory volume in one second; FVC, forced vital capacity; LLN, lower limit of normal. Severity categories defined by z-score thresholds: normal (> −1.6), mild (−1.65 to −2.5), moderate (−2.51 to −4.0), and severe (< −4.1). Percentages represent the proportion within each severity level.

### Laboratory investigations

A complete blood count (CBC) was performed for each participant. Venous blood (2–4 mL) was drawn into ethylenediaminetetraacetic acid (EDTA) tubes under aseptic conditions and analyzed immediately in laboratories accredited by the Uganda National Health Laboratory Services.

### Validity, reliability, and data quality control

Questionnaire content validity was reviewed by three independent pediatricians; a Content Validity Index ≥ 0.80 was considered acceptable (24, 25). Pretesting ensured the instrument’s reliability (Cronbach’s *α* ≥ 0.70). Spirometry reliability was maintained by adhering to the ATS/ERS calibration protocols (26, 27). The research assistants received intensive training in study protocols, ethics, and spirometry techniques. Calibration logs were completed daily, and the principal investigator conducted random spot checks and debriefing. Data were double-entered into a secure electronic database with range and consistency check. A pilot investigation was conducted and confirmed feasibility and clarity at Nile International Hospital in Jinja and Pentecostal Assemblies of God Mission Hospital in Lira.

### Data management and statistical analysis

The data were anonymized, coded, and stored on password-protected computers accessible only to the research team. Analyses were conducted using IBM SPSS version 26.

Descriptive statistics summarized baseline demographic, environmental and clinical characteristics. Prevalence of Spirometry abnormalities and distribution of spirometry patterns were presented as frequencies and percentages. Associations between independent variables and spirometry abnormalities were first explored using bivariate analyses using Chi-square or Fisher’s exact tests to obtain crude odds ratios (cORs) and 95% confidence intervals (CIs). Variables with *p* < 0.20 or clinical relevance were entered into a multivariate logistic-regression model to identify independent predictors. Multicollinearity was assessed using variance inflation factors (VIF < 5 indicating acceptable collinearity), and model fit was verified using the Hosmer-Lemeshow test. Adjusted odds ratios (aORs) and 95% CIs were reported, with *p* < 0.05 considered statistically significant. Subgroup analyses examined factors associated with specific spirometry patterns (restrictive, obstructive, and mixed). Separate logistic-regression models and interaction terms assessed potential effect modification.

### Ethical considerations

Ethical approval was obtained from the Kampala International University Research Ethics Committee (Ref. No. 2025–1738; approval date: 2 September 2025). The study was also registered with the Uganda National Council for Science and Technology (UNCST), and administrative clearance was obtained from both participating hospitals. Participation was voluntary, and individuals were free to withdraw at any stage without consequences. All study procedures adhered to the Uganda National COVID-19 and Ebola Research Guidelines and Ministry of Health safety protocols, including mask use, hand hygiene, and physical distancing, to ensure participant and staff safety. Written informed consent was obtained from parents or legal guardians after a clear explanation of the study’s objectives, procedures, potential risks, and benefits in English, Lusoga, or Lango. For illiterate caregivers, fingerprints were used in place of signatures. Children aged 7–17 years provided verbal and written assent before participation.

## Results

### Sociodemographic characteristics of participants

**Figure 1:**
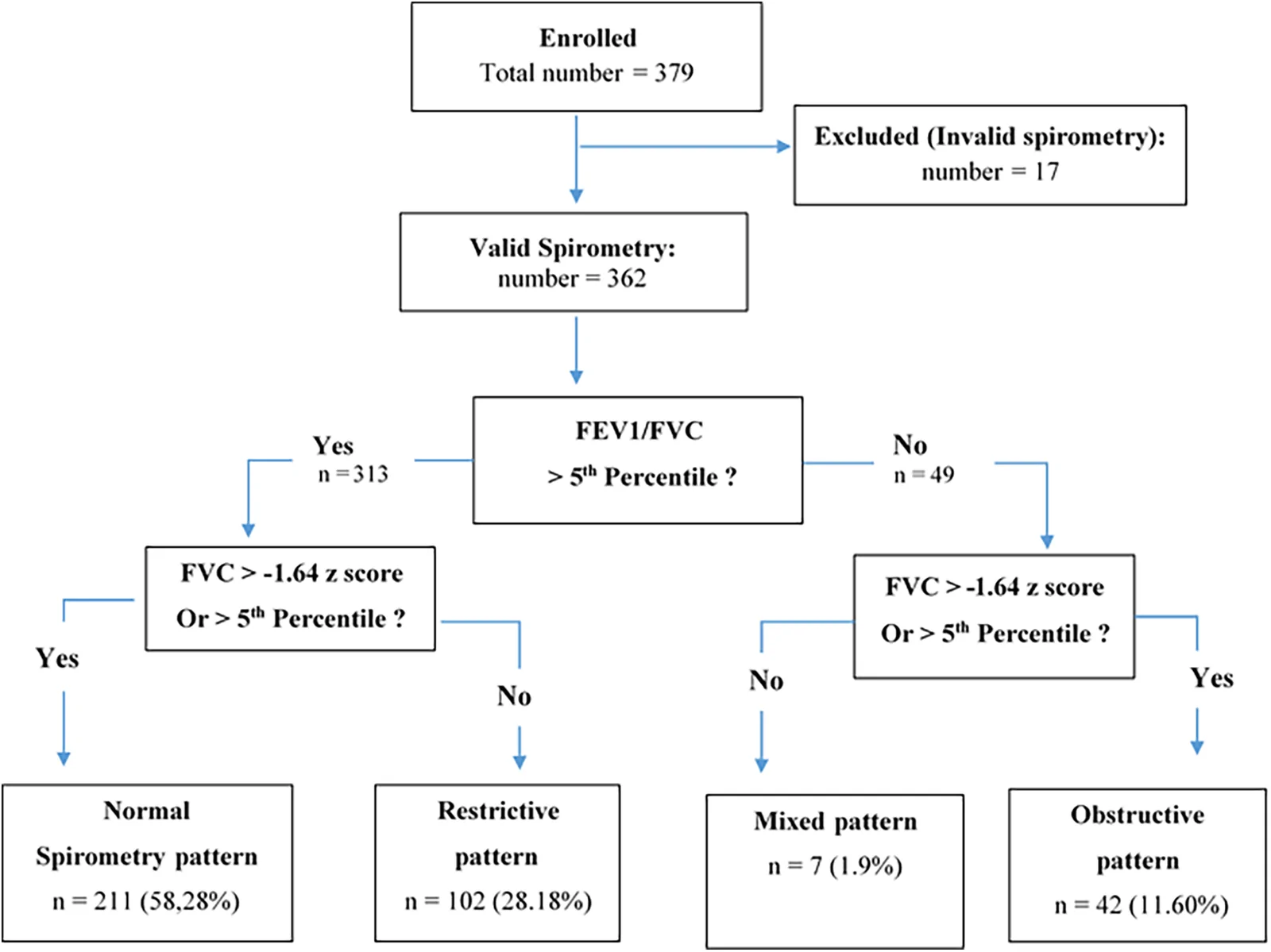
Study profile.

During the study period, 379 stable children with SCD, aged 6–18 years (target population), attended SCD specialty clinics at JRRH and LRRH. In total, 17 patients were excluded: 11 due to failure to obtain consent or assent (when required) or inability to perform the test and 6 subsequently excluded from analysis because of invalid spirometry results or incomplete data. Eligible participants were consecutively enrolled until the required sample size of 362 was reached (Figure 1).

Regarding the age distribution, 21.0% were 6–7 years, 37.8% were 8–12 years, and 41.2% were 13–18 years. There were slightly more males (54.1%) than females (45.9%). Only 5.2% reported a smoker in the household (Table 2).

**Table 2:** Sociodemographic and environmental characteristics of study participants.

Characteristic	Frequency	Percentage
Age group (years)		
6–7	76	21.0
8–12	137	37.8
13–18	149	41.2
Sex		
Male	196	54.1
Female	166	45.9
Education parent		
None	13	3.6
Primary	117	32.3
Secondary	157	43.4
Tertiary	75	20.7
Income house hold in Ugandan Shillings, UGX		
200,000	115	31.8
200–500,000	169	46.7
500,000	78	21.5
Residence		
Urban	187	51.7
Rural	175	48.3
Cooking site		
Indoor	301	83.1
Outdoor	61	16.9
Cooking fuel		
Electric	9	2.5
Firewood	200	55.2
Charcoal	129	35.6
LP gas	24	6.6
Household smoking		
No	343	94.8
Yes	19	5.2
Active smoke		
No	360	99.4
Yes	2	0.6

UGX, Ugandan Shillings; LP gas, liquefied petroleum gas.

### Clinical and laboratory characteristics

In this study, 44 participants (12.2%) had not completed routine immunization, including 28 (7.7%) who had not received all recommended doses of the pneumococcal conjugate vaccine (PCV). Approximatively 32.6% of participants were underweight, and 82.9% were receiving hydroxyurea therapy (Table 3).

**Table 3:** Clinical and laboratory characteristics of study participants .

Characteristic	Frequency	Percentage
Routine immunization		
Incomplete	44	12.2
Complete	318	87.8
PCV recommended dose		
No	28	7.7
Yes	334	92.3
Covid-19 vaccine		
No	358	98.9
Yes	4	1.1
Nutrition status		
Healthy	237	65.5
Underweight	118	32.6
Overweight	7	1.9
Previous admissions		
None	135	37.3
1–5	185	51.1
6–10	34	9.4
>10	8	2.2
Previous ACS		
None	295	81.5
1 episode	56	15.5
2 + episodes	11	3.0
Blood transfusion		
None	108	29.8
1	131	36.2
2–5	105	29.0
>5	18	5.0
Hydroxyurea		
No	62	17.1
Yes	300	82.9
Hemoglobin level		
10 g/dL +	120	33.1
7–9.9	199	55.0
5–6.9	42	11.6
<5	1	0.3

ACS, acute chest syndrome; PCV, pneumococcal conjugate vaccine.

### Prevalence and patterns of spirometry abnormalities

**Figure 2:**
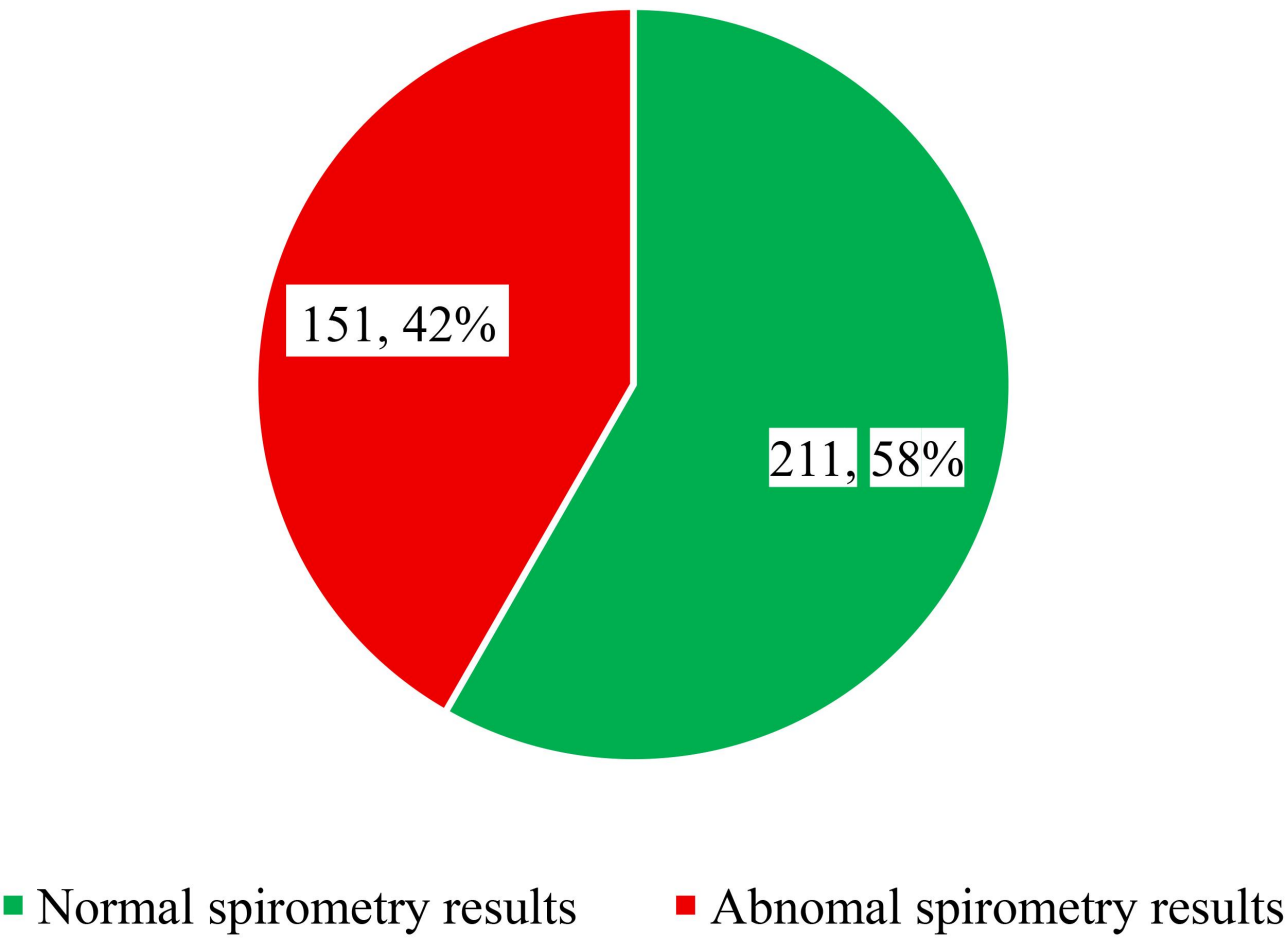
Prevalence of spirometry abnormalities among children with sickle cell Anemia in northern and eastern Uganda.

**Figure 3:**
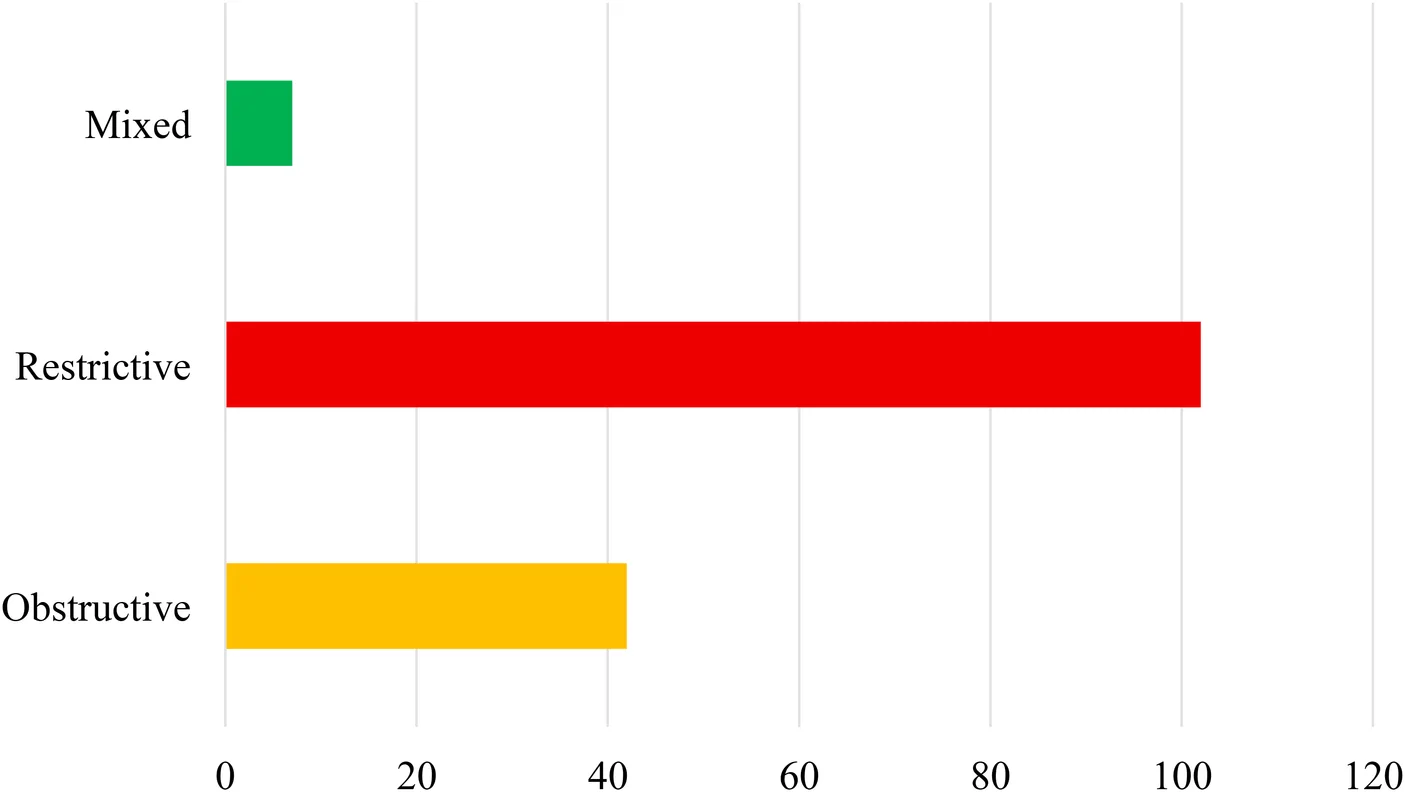
Patterns of spirometry abnormalities among children with sickle cell Anemia in northern and eastern Uganda.

Of the 362 participants enrolled in this study, abnormal spirometry results were observed in 151, representing a prevalence of 41.7% with a 95% confidence interval of 36.7%–47.0% (Figure 2). Of the 151 that were found to have abnormal spirometry, majority had a restrictive pattern seen in 102 (67.5%), followed by an obstructive type seen in 42 (27.8%), with the least represented being a mixed pattern which was only seen in 7 (4.6%) as depicted in Figure 3.

### Factors associated with of spirometry abnormalities

Multivariate logistic regression was conducted to assess and identify factors that are independently and significantly associated with the odds of spirometry abnormalities among Children with SCD.

The variables considered for multivariable analysis (*p* < 0.2) were: age category, sex, residence, cooking site, recommended PCV vaccine dose, Nutritional status, previous admission, previous acute chest syndrome (ACS) and hemoglobin level. In the multivariable analysis, the independent factors for spirometry abnormalities among children with SCD attending JRRH and LRRH were: increasing age (aOR=3.346, CI = 1.375–8.142, *P* = 0.008) for age 8–12 years and (aOR=13.532, CI = 5.687–32.201, *P* < 0.001) for 13–18 years, not receiving all the recommended PCV dose (aOR=3.474, CI = 1.618–9.153, *P* = 0.022), being underweight (aOR=3.871, CI = 2.189–6.845, *P* < 0.001) and previous history of ACS, with (aOR=1.658, CI = 1.035–3.393, *P* = 0.045) for 1 episode and (aOR=3.295, CI = 1.016–14.526, *P* = 0.044) for more than 1 episode. The odds for spirometry abnormalities were increased by over three times among those aged between 8 and 12 years, among those who did not receive the recommended PCV vaccine dose, those who were under weight and those with more than 1 previous episode of ACS. For those who were aged 13–18 years, the odds for abnormal spirometry indices were increased by over 13 times, while the odds were increased by close to two times among those who had one previous episode of ACS (Table 4).

**Table 4:** Bivariate and multivariable analysis of factors associated with for spirometry abnormalities among children with sickle cell disease in northern and eastern Uganda.

Characteristic		Bivariable analysis			Multivariable analysis	
	cOR	95% CI	*P* value	aOR	95% CI	*P* value
Age group (years)						
6–7	Ref					
8–12	3.888	1.718–8.799	0.001	3.346	1.375–8.142	0.008
13–18	17.347	7.729–38.931	<0.001	13.532	5.687–32.201	<0.001
Sex						
Male	Ref					
Female	1.426	0.937–2.171	0.098	1.582	0.911–2.747	0.103
Residence						
Urban	1.441	0.946–2.194	0.089	1.399	0.810–2.415	0.229
Rural	Ref					
Cooking site						
Indoor	0.642	0.369–1.115	0.115	0.636	0.330–1.226	0.117
Outdoor	Ref					
PCV recommended						
No	3.874	1.658–9.053	0.002	3.474	1.618–9.153	0.022
Yes	Ref					
Nutrition status						
Healthy	Ref					
Underweight	4.642	2.893–7.450	<0.001	3.871	2.189–6.845	<0.001
Overweight	N/A					
Previous admissions						
None	Ref					
1–5	0.833	0.530–1.310	0.429	0.687	0.388–1.217	0.198
>5	1.825	0.906–3.675	0.092	1.321	0.535–3.263	0.547
Previous ACS						
None	Ref					
1 episode	1.858	1.045–3.303	0.035	1.658	1.035–3.393	0.045
2 + episodes	4.295	1.116–16.526	0.034	3.295	1.016–14.526	0.044
Hemoglobin level						
10 g/dL +	Ref					
7–9.9	1.217	0.765–1.937	0.406	1.317	0.712–2.438	0.381
<7	1.746	0.865–3.526	0.120	2.053	0.851–4.953	0.109

cOR, crude odds ratio; aOR, adjusted odds ratio; CI, confidence interval; PCV, pneumococcal conjugate vaccine.

Additionally, we conducted multivariate subgroups analysis, to assess the independent factors associated with each pattern of spirometry abnormalities.

The independent factors that increased the odds of restrictive spirometry pattern among children with SCA attending JRRH and LRRH were: age 8–12 years (aOR=4.176, CI = 1.386–12.579, *P* = 0.011), age 13–18 years (aOR=15.961, CI = 5.510–46.236, *P* < 0.001) and not receiving all the routine and recommended PCV doses (aOR=3.974. CI = 1.7189.253, *P* = 0.031), and being underweight (aOR=3.810, CI = 1.984–7.317, *P* < 0.001) as detailed in Table 5.

**Table 5:** Subgroup multivariate analysis of factors for restrictive spirometry pattern children with sickle cell disease in northern and eastern Uganda.

Characteristic		Multivariable analysis	
	aOR	95% CI	*P* value
Age group (years)			
6–7	Ref		
8–12	4.176	1.386–12.579	0.011
13–18	15.961	5.510–46.236	<0.001
Sex			
Male	Ref		
Female	1.607	0.853–3.027	0.142
Residence			
Urban	1.743	0.926–3.281	0.085
Rural	Ref		
Cooking site			
Indoor	0.374	0.182–1.767	0.070
Outdoor	Ref		
PCV recommended dose			
No	3.974	1.718–9.253	0.031
Yes	Ref		
Nutrition status			
Healthy	Ref		
Underweight	3.810	1.984–7.317	<0.001
Overweight	N/A		
Previous admissions			
None	Ref		
1–5	0.552	0.293–1.042	0.067
>5	0.537	0.173–1.663	0.281
Previous ACS			
None	Ref		
1 episode	1.358	0.835–3.393	0.110
2 + episodes	2.895	0.936–14.526	0.104
Hemoglobin level			
10 g/dL +	Ref		
7–9.9	1.337	0.680–2.630	0.400
<7	1.918	0.673–5.467	0.223

aOR, adjusted odds ratio; CI, confidence interval; PCV, pneumococcal conjugate vaccine.

The independent factors that increased the odds for obstructive Spirometry pattern among Children with SCD attending JRRH and LRRH were: age 13–18 years (aOR=15.475, CI = 3.166–75.654, *P* = 0.001), not receiving recommended PCV dose (aOR=4.474, CI = 2.618–8.153, *P* = 0.032), being underweight (aOR=5.220, CI = 2.039–13.367, *P* = 0.001), having more than 5 previous admissions (aOR=6.293, CI = 1.595–24.827, *P* = 0.009) and having a hemoglobin level below 7 g/dL (aOR=4.020, CI = 1.026–15.761, *P* = 0.046) as detailed in Table 6. However, because this subgroup analysis was based on only 42 obstructive cases, these findings must be interpreted with caution. These specific subgroup associations should be considered hypothesis-generating rather than definitive.

**Table 6:** Subgroup multivariate analysis of factors for obstructive spirometry pattern among children with sickle cell disease in northern and eastern Uganda .

Characteristic		Multivariable analysis	
	aOR	95% CI	*P* value
Age group (years)			
6–7	Ref		
8–12	3.333	0.657–16.898	0.146
13–18	15.475	3.166–75.654	0.001
Sex			
Male	Ref		
Female	2.077	0.806–5.350	0.130
Residence			
Urban	1.458	0.593–3.583	0.412
Rural	Ref		
Cooking site			
Indoor	3.660	0.881–15.212	0.074
Outdoor	Ref		
PCV recommended dose			
No	4.474	2.618–8.153	0.032
Yes	Ref		
Nutrition status			
Healthy	Ref		
Underweight	5.220	2.039–13.367	0.001
Overweight	N/A		
Previous admissions			
None	Ref		
1–5	1.085	0.422–2.787	0.866
>5	6.293	1.595–24.827	0.009
Previous ACS			
None	Ref		
1 episode	1.958	0.935–4.393	0.055
2 + episodes	2.295	0.916–7.526	0.054
Hemoglobin level			
10 g/dL +	Ref		
7–9.9	1.088	0.360–3.284	0.881
<7	4.020	1.026–15.761	0.046

aOR, adjusted odds ratio; CI, confidence interval; PCV, pneumococcal conjugate vaccine.

The sub group analysis for mixed abnormal PFTs could not yield any meaningful models due to the small number of observations (only 7 participants were found to have mixed mixed spirometry pattern).

## Discussion

This study provides new evidence of the burden and characteristics of spirometry abnormalities among children and adolescents with homozygous SCD (HbSS) in eastern and northern Uganda. The prevalence of spirometry abnormalities was remarkably high, demonstrating that chronic pulmonary dysfunction is already well-established during childhood in these settings.

Although both Jinja and Lira regional referral hospitals provide dedicated SCD clinics and preventive care services, structured respiratory surveillance is not consistently implemented in routine practices.

Variability in adherence to hydroxyurea and prophylactic measures, influenced by cost, drug availability, and caregiver perceptions, also contributes to cumulative disease related end-organ injury over time.

This observation is concerning because the study population was enrolled in a clinically stable steady state, suggesting that pulmonary injury in SCD may progress silently, long before obvious chronic respiratory manifestations emerge.

Methodologically, this study focused exclusively on children with homozygous SCD (HbSS), the most clinically severe SCD genotype, which likely increased the observed frequency of Spirometry Abnormalities.

Even when clinically stable, children with HbSS experience persistent hemolysis, chronic anemia, and recurrent vaso-occlusive microinfarcts of the alveolar-capillary interface.

By applying ATS/ERS 2019 spirometry standards and GLI-2012 reference equations validated for sub-Saharan African populations, subtle or subclinical decline was captured with high diagnostic precision, yielding a more accurate representation of early impairment (23, 28, 29). The use of non-local GLI-2012 reference equations may have influenced prevalence estimates and represents a limitation of this study. Development of Ugandan-specific reference equations is warranted (22).

This important prevalence is consistent with another recent study at the Mulago National Referral Hospital in Kampala, with a strong Confidence Interval overlap (15). Both point estimates from Uganda are broadly consistent with several African studies from lower or middle income countries (LMICs), such as, Nigeria, the Democratic Republic of the Congo (DRC), and South Africa (16, 30, 31). These studies indicate a broadly consistent burden of abnormal PFTs across diverse African settings, likely reflecting shared exposures such as recurrent respiratory infections, and variability in access to comprehensive SCD care.

However, in contrast, our prevalence was slightly higher than that reported in Kenya and Egypt (32, 33). These lower values may reflect differing study populations, including variations in socioeconomic levels, environmental exposures or differences in spirometry reference standards and technician expertise may also contribute to measurement heterogeneity across settings.

Overall, despite numerical variability, the African evidence converges around a high burden of abnormal PFTs among children with SCD, with our estimate positioned at the upper margin of this range. The high prevalence in LMICs may therefore be attributed to multiple regional factors such as recurrent pulmonary infections, under-nutrition, suboptimal vaccination coverage, and limited access to routine pulmonary screening.

When compared with studies from high-income settings, the prevalence of abnormal PFTs observed in our study falls within the broad international range but reveals meaningful differences that reflect systemic and contextual factors.

Our findings are statistically consistent with those from Bahrain and partially overlap with those of a study from United Kingdom (UK), suggesting a comparable overall burden despite significant differences in healthcare access, early diagnosis, and preventive interventions (19, 34).

In the current study the restrictive spirometry pattern predominated, accounting for more than two-thirds of all abnormal results. This pattern which reflects possible underlying restrictive ventilatory defects, is consistent with the dominant parenchymal pathophysiology of SCD, in which repetitive microvascular occlusion and inflammatory insult promote alveolar fibrosis, reduced lung compliance, and diminished total lung capacity (13).

The exclusion of children with known asthma or chronic respiratory disease likely reduced the proportion of obstructive pattern, revealing the true parenchymal burden.

A previous Ugandan study by Aol et al. (15) similarly demonstrated that more than half of the children with abnormal lung function had a restrictive pattern in Kampala (15). Comparable restrictive predominance has been reported in several African studies: in Nigeria, Malawi, and the DRC (16, 21, 30).

In contrast, high-income settings tend to demonstrate more diverse distributions, often with stronger obstructive representation. Two studies from the United States (USA) in Alabama and Michigan reported more cases of obstructive pattern than restrictive Pattern (35, 36); however these studies did not exclude asthmatic children. In a study from UK, obstructive pattern was four times more frequent (34).

Middle-income studies show similar heterogeneity, for instance, studies from Bahrain and Brazil reported near-equal restrictive and obstructive patterns (18, 19).

This sharp contrast suggests that repeated infection burden, biomass fuel exposure, nutritional deficits, and incomplete access to hydroxyurea, transfusion support, and pulmonary rehabilitation likely accelerate parenchymal injury in African children leading to restrictive patterns manifesting earlier.

Several factors were independently associated with abnormal spirometry results in the present study. Increasing age showed a strong gradient: children aged 8–12 years had approximately threefold higher odds of spirometry abnormality, while adolescents aged 13–18 years had more than thirteenfold higher odds than younger children.

This supports a cumulative-injury model in which silent microvascular damage accumulates throughout childhood.

Compelling longitudinal evidence reinforces the interpretation that pulmonary function abnormalities in SCD begin early and worsen progressively with age.

The landmark Canadian cohort, in Toronto, demonstrated a striking age-related shift in patterns with restrictive defects increasing almost ten-fold over adolescence (37).

Similarly, an investigation in London reported that the steepest annual decline in lung volumes occurred during early childhood, with the early cohort (shorter follow-up) showing faster deterioration than the late-adolescent cohort (longer follow-up), indicating that the greatest velocity of pulmonary decline occurs in the first decade of life rather than late adolescence (38).

These data strongly support the concept that early lung-function impairment in SCD is not transient, but marks the beginning of a chronic physiological deterioration trajectory that must be intercepted before adolescence to prevent permanent loss of lung capacity (37–39).

We found that undernutrition emerged as a major determinant (fourfold increased odds), aligning with evidence from an investigation in Kampala reported that wasting nearly doubled the risk of abnormal spirometry, while the study in DRC found that children with low body-mass-index (BMI) z-scores were four times more likely to have restrictive patterns (15, 16). Emerging evidence supports that chronic malnutrition impairs lung growth, reduces alveolar surface area, weakens respiratory muscles, and increases susceptibility to severe respiratory infections all of which predispose patients to long-term respiratory morbidity (36, 40).

Incomplete pneumococcal vaccination and a history of ACS were also strongly associated with abnormal PFTs, supporting the concept that recurrent lower respiratory tract infections and vaso-occlusive events are not merely isolated crises but lead to structural insults with lasting consequences.

It is now well established that chronic pulmonary sequelae such as SCD-associated pulmonary hypertension and pulmonary fibrosis are major determinants of long-term prognosis in SCD, contributing to exercise intolerance, chronic dyspnea, impaired quality of life, and premature mortality in adulthood (41, 42).

Importantly, restrictive impairment in childhood may represent the earliest detectable stage of a cardiopulmonary continuum that culminates in pulmonary fibrosis and pulmonary hypertension in later life both of which are major causes of mortality in SCD (5, 13).

Progressive alveolar-capillary injury, microvascular thrombosis, and chronic hemolysis contribute to progressive parenchymal stiffening and vascular remodeling (13).

Confirming these advanced complications requires echocardiography, high-resolution computed tomography (HRCT) and, ultimately, right-heart catheterization all of which are high-cost and rarely accessible in resource-limited settings (43).

Therefore, spirometry represents the only practical, scalable tool capable of signaling early chronic lung disease in children before irreversible cardiopulmonary remodeling becomes clinically apparent (29).

Taken together, these findings indicate that chronic pulmonary injury begins early in.

Ugandan children with SCD, progresses silently, and is driven by modifiable risk factors.

Addressing these modifiable factors including vaccination completion, nutritional support, and aggressive ACS prevention and treatment coupled with the integration of periodic spirometry surveillance, could substantially alter respiratory trajectories and reduce long-term cardiopulmonary morbidity in this high-risk population.

While hydroxyurea therapy is widely recognized to improve overall clinical outcomes and reduce acute chest syndrome (ACS) episodes in sickle cell disease, our analysis did not demonstrate a statistically significant association between hydroxyurea use and spirometry abnormalities. This lack of association may be due to limitations in our data collection, as we did not comprehensively capture details on treatment duration, precise dosage, or medication adherence. Future prospective trials are required to fully elucidate the longterm impact of optimized hydroxyurea therapy on pediatric lung function trajectories in Sub-Saharan Africa.

Although the recent Kampala study provided valuable initial evidence of lung-function impairment among urban children with SCD in Uganda, its findings were derived from a single urban center and therefore did not capture the broader spectrum of disease across other high-burden regions (15). Northern and eastern Uganda differ substantially from Kampala in terms of socio-economic vulnerability, nutritional status, environmental exposure, and access to preventive services, all of which are potential determinants of respiratory outcomes in SCD. To date, no multicenter assessment has been conducted in Uganda to evaluate the burden and patterns of spirometry-defined abnormalities across different geographical and population profiles in Uganda. Therefore, this study therefore provides the first regional, multi-institutional data on the prevalence and factors associated with spirometry abnormalities among children with confirmed HbSS in Eastern (Jinja) and Northern (Lira) Uganda.

## Conclusion

The findings from this study reinforce the evidence that spirometry-defined abnormalities areis common and early onset, among children and adolescents with sickle cell disease.

The prevalence of spirometry abnormalities among children attending the two selected hospitals in Uganda is high, such as in every five patients, with two out of five patients having abnormal spirometry results, with the restrictive type being the most common pattern.

Most affected individuals are clinically asymptomatic at the time of assessment, indicating that reliance on symptoms alone substantially underestimates the true burden of pulmonary morbidity.

Increasing age, undernutrition, incomplete immunization against pneumococcus, and recurrent acute chest syndrome were significantly and independently associated with spirometry abnormalities.

### Study strengths and limitations

The Multicenter design across two tertiary hospitals, enhances generalisability within eastern and northern Uganda, among regions with a high prevalence of SCD. The use of technical and interpretative standards and validated tools (ATS/ERS guidelines and GLI12 reference equation) for spirometry testing, ensures methodological robustness.

Finally, the inclusion of a wide range of socio-demographic and clinical variables enabled rigorous multivariate analysis and identification of modifiable risk factors.

However, this study has a limitation: the cross-sectional design precluded causal inference between exposures and outcomes. In addition, advanced pulmonary investigations such as plethysmography, diffusion capacity testing, and high-resolution computed tomography (HRCT) were not available, limiting the assessment of structural and functional lung abnormalities. Consequently, restrictive lung-function impairment was identified using spirometry-based criteria (low FVC with preserved FEV1/FVC ratio) rather than direct lung volume measurements. Therefore, true restrictive ventilatory defects could not be definitively confirmed with total lung capacity (TLC) measurements, which were unavailable in our setting.

### Recommendations for clinical practice, research priorities and public health perspectives

− Routine spirometry should be integrated into standard SCD follow-up (at least annually from age 6 years) and after each ACS episode to detect and monitor restrictive changes early.
− Strengthening preventive measures through the full completion of pneumococcal vaccination schedules, nutritional support, anemia control, consistent hydroxyurea use, and infection prevention is crucial.
− Equip and train healthcare facilities particularly regional referral hospitals, with spirometers and staff skilled in ATS/ERS-compliant testing and interpretation.
− Promote community awareness of vaccination adherence, nutrition, and prompt careseeking for respiratory symptoms through school and caregiver engagement.
− Longitudinal and Interventional studies are needed to track pulmonary function trajectories and evaluate interventions such as hydroxyurea, physiotherapy (incentive spirometry), and nutritional support.

## Data Availability

The datasets presented in this study can be found in online repositories. The names of the repository/repositories and accession number(s) can be found in the article/Supplementary Material.
