# Supplementary material for: Prevalence, patterns and factors associated with spirometry abnormalities among children and adolescents with sickle cell disease in northern and eastern Uganda: a cross-sectional study
Source: Front Pediatr. 2026 Aug 28;14:1891576. doi: 10.3389/fped.2026.1891576 (PMC13562054; doi:10.3389/fped.2026.1891576)
Supplement: Supplementary file 1 [file Table2.docx]

| **Variable Values** | | |
| --- | --- | --- |
| Value | | Label |
| Hospital | 1 | JRRH |
|  | 2 | LRRH |
| Age_group | 1 | 6-7 |
|  | 2 | 8-12 |
|  | 3 | 13-18 |
| Sex | 1 | Male |
|  | 2 | Female |
| Tribe | 1 | Musoga |
|  | 2 | Langi |
|  | 3 | Others |
| Parent_education | 0 | None |
|  | 1 | Primary |
|  | 2 | Secondary |
|  | 3 | Tirtiary |
| Religion | 0 | Other |
|  | 1 | Christian |
|  | 2 | Muslim |
| Occupation | 1 | Formal occupation |
|  | 2 | Busness |
|  | 3 | House wife |
|  | 4 | Farmer/peasant |
| Household_income | 1 | <200k |
|  | 2 | 200-500K |
|  | 3 | >500K |
| Residence | 1 | Urban |
|  | 2 | Rural |
| Cooking_site | 1 | Indoor |
|  | 2 | Outdoor |
| Cooking_fuel | 0 | Electric |
|  | 1 | Firewood |
|  | 2 | Charchol |
|  | 3 | Gas |
| Household_smoking | 0 | No |
|  | 1 | Yes |
| Lighting_type | 0 | Electricity/solar |
|  | 1 | Fuel based |
| Active_smoke | 0 | No |
|  | 1 | Yes |
| Routine_immunization | 0 | Incomplete |
|  | 1 | Complete |
| PCV_extra_doses | 0 | No |
|  | 1 | Yes |
| COVID_vaccine | 0 | No |
|  | 1 | Yes |
| Nutrition_status | 0 | Healthy |
|  | 1 | Underweight |
|  | 2 | Overweight |
| Prevous_admissions | 0 | None |
|  | 1 | 1-5 |
|  | 2 | 6-10 |
|  | 3 | >10 |
| Prevous_ACS | 0 | None |
|  | 1 | 1 episode |
|  | 2 | 2+ episodes |
| Prevous_Painful_crisis | 0 | None |
|  | 1 | 1 episode |
|  | 2 | 2+ episodes |
| Blood_transfusion | 0 | None |
|  | 1 | 1 |
|  | 2 | 1-5 |
|  | 3 | >5 |
| Hydroxyurea_treatment | 0 | No |
|  | 1 | Yes |
| Chronic_cough | 0 | No |
|  | 1 | Yes |
| Recurrent_wheeze | 0 | No |
|  | 1 | Yes |
| Sleep_breathing_disorder | 0 | No |
|  | 1 | Yes |
| Cyanosis | 0 | No |
|  | 1 | Yes |
| Digital_clubbing | 0 | No |
|  | 1 | Yes |
| Hb_level_group | 1 | 10g/dl+ |
|  | 2 | 7-9.9 |
|  | 3 | 5-6.9 |
|  | 4 | <5 |
| Lung_function | 0 | Normal |
|  | 1 | Abnormal |
| Lung_pattern | 0 | Normal |
|  | 1 | Obstructive |
|  | 2 | Restrictive |
|  | 3 | Mixed |
| CSR_HRQoL_Interpretation | 0 | Normal QOL |
|  | 1 | Poor QOL |
| PPR_QoL_Interpretation | 0 | Normal QOL |
|  | 1 | Poor QOL |
| Childphysical | 1 | < 69.7 |
|  | 2 | 69.7+ |
| Childemotional | 1 | < 69.7 |
|  | 2 | 69.7+ |
| Childsocial | 1 | < 69.7 |
|  | 2 | 69.7+ |
| Childphyscological | 1 | < 69.7 |
|  | 2 | 69.7+ |
| Parentphysical | 1 | < 65.4 |
|  | 2 | 65.4+ |
| Parentemotional | 1 | < 65.4 |
|  | 2 | 65.4+ |
| Parentsocial | 1 | < 65.4 |
|  | 2 | 65.4+ |
| Parentphyc | 1 | < 65.4 |
|  | 2 | 65.4+ |
